# Assessing complex interventions: a systematic review of outcomes used in randomised controlled trials on STI partner notification in high-income countries

**DOI:** 10.1186/s12889-023-16763-9

**Published:** 2023-09-21

**Authors:** Victoire Sawras, Sylvie Deuffic-Burban, Marie Préau, Bruno Spire, Yazdan Yazdanpanah, Karen Champenois

**Affiliations:** 1 Université Paris Cité and Université Sorbonne Paris Nord, Inserm, IAME, Paris, F-75018 France; 2 Institut de Psychologie, Université Lumière Lyon 2, Inserm, U1296, Lyon, France; 3 Aix Marseille Univ, Inserm, IRD, SESSTIM, ISSPAM, Marseille, France; 4 Service de maladies infectieuses et tropicales, Hôpital Bichat Claude Bernard, Paris, F-75018 France; 5 Inserm IAME – Faculté de Médecine Bichat, 16 rue Henri Huchard, Paris, 75018 France

**Keywords:** Complex interventions, Randomised controlled trials, Outcomes, Partner notification, STI, HIV

## Abstract

**Background:**

Partner notification interventions are complex and assessing their effectiveness is challenging. By reviewing the literature on the effectiveness of partner notification interventions, our aim was to evaluate the choice, collection, and interpretation of outcomes and their impact on study findings.

**Methods:**

We conducted a systematic review of individual-level randomised controlled trials evaluating the effectiveness of partner notification interventions for bacterial STIs, HIV or sexually transmitted HCV in high-income countries since 2000. Partner notification interventions included assisted patient referral interventions and expedited treatment. The content analysis was carried out through a narrative review.

**Results:**

In the 9 studies that met the inclusion criteria, 16 different outcomes were found. In most studies, one or two outcomes assessing partner notification practices were associated with an outcome reflecting STI circulation through index case reinfections. These outcomes assessed the main expected effects of partner notification interventions. However, partner notification is composed of a succession of actions between the intervention on the index case and the testing and/or treatment of the notified partners. Intermediate outcomes were missing so as to better understand levers and barriers throughout the process. Potential changes in participants’ sexual behaviour after partner notification, e.g. condom use, were outcomes reported in only two studies assessing interventions including counselling. Most outcomes were collected through interviews, some weeks after the intervention, which might lead to desirability and attrition biases, respectively. Assessment of the effectiveness of partner notification interventions on partner testing/treatment was limited by the collection of data from index cases. Few data describing index cases and their partners were provided in the studies. Additional data on the number and type of exposed partners and the proportion of partners already aware of their infection before being notified would help to interpret the results.

**Conclusions:**

These insights would help to understand why and under what conditions the intervention is considered effective and therefore can be replicated or adapted to other populations and contexts.

**Supplementary Information:**

The online version contains supplementary material available at 10.1186/s12889-023-16763-9.

## Introduction

Since 2000, sexually transmitted infections (STIs) have been rising in France, as in most high-income countries, with an increase in gonorrhoea and chlamydia, a resurgence of syphilis, and the emergence of lymphogranuloma venereum (LGV) [1–6]. Men who have sex with men (MSM), especially those living with HIV or using PrEP, are particularly affected by STIs, followed by heterosexual men and women with multiple sexual partners [2–12]. As these epidemics are concentrated in relatively small highly sexually active populations, an intervention to increase testing and treatment of STI-exposed sexual partners could break transmission chains in limiting onward transmission [13].

Partner notification interventions incite people diagnosed with STIs to inform their past and current exposed sexual partners, encourage them to get tested, and/or access treatment or prevention. There are two approaches for partner notification: (1) patient referral: index cases are encouraged to inform their sexual partners themselves about their STI exposure, which can be enhanced with additional support or tools, and (2) provider referral: a trained provider assists the consenting index cases in notifying their partners on their behalf [14]. Expedited partner treatment where the index case delivers a treatment or a prescription for treatment to their partners without medical examination or testing, can also be considered as part of partner notification interventions [15].

Some countries like the United States, the United Kingdom or the Netherlands have for several years proposed partner notification services for HIV or STIs, including expedited partner treatment, mainly in sexual health centres or STI clinics [16–20]. Such an approach, offered routinely at the time of STI diagnosis, has not been implemented in France and many countries yet. Few systematic reviews have evaluated the effectiveness of partner notification interventions for STIs including HIV [21–23]. They highlighted no single optimal strategy regardless of the STI. However, enhanced patient referral with written information and expedited partner treatment were more effective than simple patient referral [21, 22]. Provider referral interventions were evaluated for HIV partner notification only and have been shown to be effective [23]. Even if not all exposed partners were notified, the authors generally found a higher proportion of partners informed of their STI exposure in the intervention group than in the control group and, when data were available, a high STI prevalence among notified partners who get tested after being notified [23]. These reviews considered studies from both high-income countries and countries with limited resources, including partner notification contexts specific to the latter in terms of populations, cultures, STI prevalence and therefore proposed interventions. A review focused on high-income countries would help to inform decision-makers about interventions that could be implemented in testing centres, where a large proportion of STIs are diagnosed [24]. Furthermore, these reviews highlighted some limitations in summarising the evidence on partner notification effectiveness due to the differences in partner notification interventions, the diversity of outcomes and the way outcomes were reported.

Evaluating the effectiveness of partner notification interventions is challenging because they are complex interventions as defined by the Medical Research Council [25–27]. From an intervention, the partner notification process relies on a combination of several components. These components involve different parties (index cases, their sexual partners, partner notification providers); each stakeholder has a role to play in making the process work with impacts at several levels: individual (health benefits) and community level (decreased incidence rates). The complexity of interventions also lies in the interaction between the components of the intervention and contextual factors (e.g. individual, organisational, social, cultural) [25–27]. Evaluating such interventions, therefore, requires complementary outcomes whose relevance is crucial.

The diversity of interventions and methods used to assess them has been highlighted in the reviews on the topic as a limitation to drawing conclusions about the effectiveness of partner notification. The use of a large number of outcomes is consistent with the evaluation of a complex intervention. However, most of these outcomes assessed the later steps of the partner notification process.

To ease the evaluation and comparison of partner notification interventions, we assessed the choice, collection, and interpretation of outcomes from randomised controlled trials evaluating the effectiveness of partner notification interventions for STIs and their effect on study findings. For this, a systematic review was conducted focusing on randomised controlled trials evaluating the effectiveness of partner notification interventions for STIs in high-income countries since 2000, the year of STI increase was first reported in Europe [28]. The review focused on individual-level randomised controlled trials in order to assess standardised interventions that could be replicated at the individual-level if proven effective.

## Methods

This systematic review was conducted and reported according to the Cochrane and PRISMA guidelines, respectively [29, 30]. The review was not registered in PROSPERO.

We searched electronic databases (Medline, Embase, Cochrane Library that also covers ClinicalTrials.gov (CT.gov) and the International Clinical Trials Registry Platform (ICTRP), Web of Science and the Cumulated Index to Nursing and Allied Health Literature (CINAHL)) from January 2000 to December 2021. The search strategy covered controlled terms and free text words on specific sexually transmitted infections combined with partner notification and randomised controlled trial: (STI OR HIV OR HCV) AND (partner notification OR partner treatment) AND randomised controlled trial. Full search strategies for all databases are presented in Additional file 1. The websites of international infectious disease conferences focusing on HIV and STI, and the reference lists of the retrieved articles were checked for additional studies published over the same time period.

We included in the analysis randomised controlled trials designed to assess the effectiveness of partner notification interventions at individual-level for bacterial STIs (chlamydia, gonorrhoea, syphilis), HIV or sexually transmitted HCV in high-income countries. Partner notification interventions included patient referral, provider referral, expedited partner treatment or any other intervention aiming to enhance notification, testing or treatment of exposed partners.

**Fig. 1:**
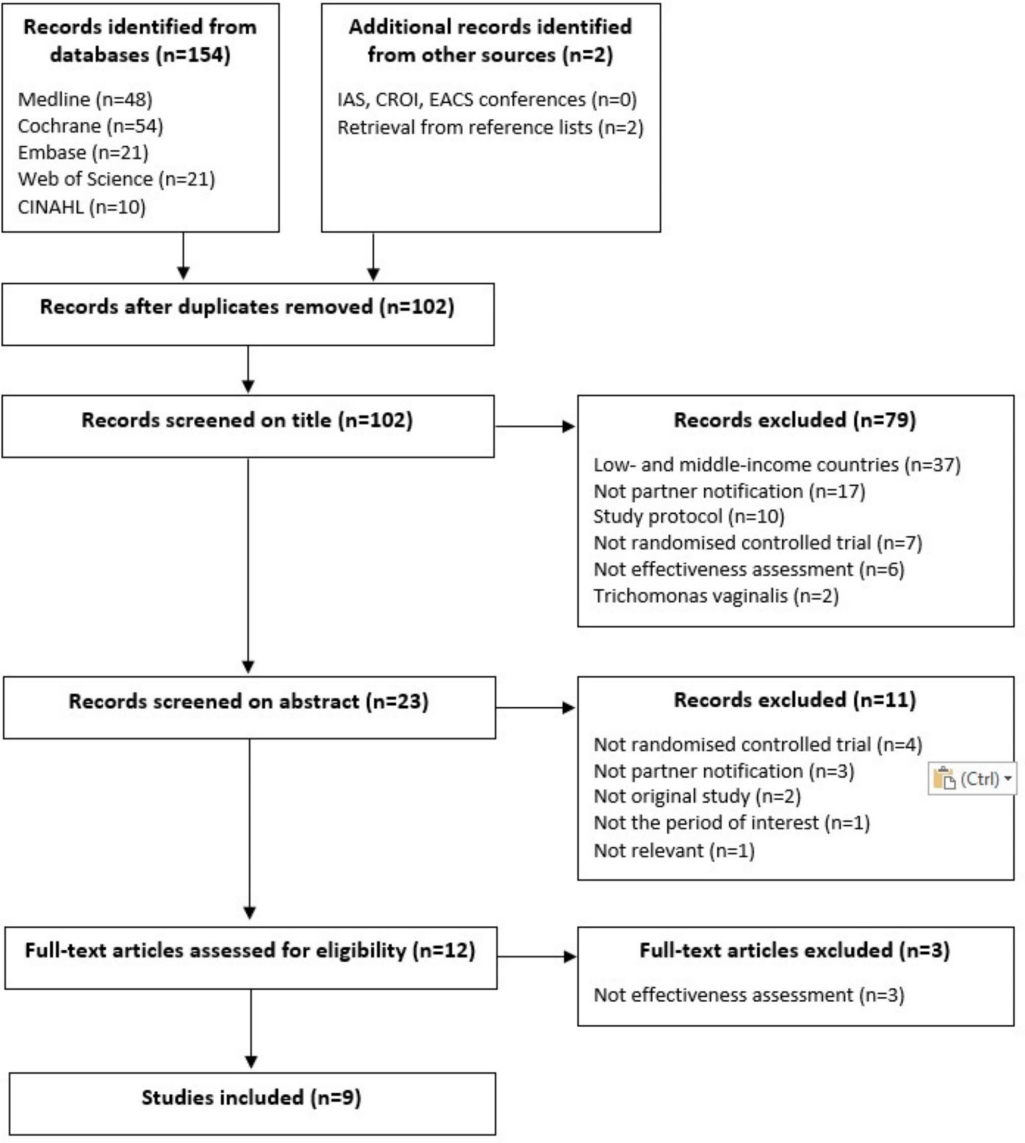
Flow chart of selection and inclusion of articles in the review

We excluded trials conducted in low- and middle-income countries, randomised controlled trials assessing the effectiveness of partner notification at population-level (stepped-wedge randomised trials, cluster randomised trials) and study protocols. The randomised controlled trials that failed to assess the effectiveness of partner notification, and finally reported no effectiveness results were excluded (Fig. 1). Only studies published in English or French were retained in the review.

References were screened based on titles and abstracts by one reviewer. When eligibility could not be assessed at this stage, full texts were read. In the case of any uncertainty in the selection and inclusion of articles, the decision was made by mutual consensus with a second reviewer.

The full texts of the included references were read and data were extracted by one reviewer using a standardised form. Extracted data included settings, participants, interventions, objectives, outcomes, main results, identified biases and any relevant comments. The extracted data were reviewed by a second reviewer to ensure that all relevant data had been collected.

The revised Cochrane risk of bias tool (RoB 2) was used by one reviewer to assess the risk of bias of the outcomes used in the randomised controlled trials included, helped to identify biases and contributed to the analysis [31]. A content analysis was conducted on the selected studies to explore which outcomes were chosen with regard to the interventions and populations, how they were collected and which factors influenced their interpretation.

## Results

### Study selection

One hundred and sixty-six references were found and nine randomised controlled trials assessing the effectiveness of STI partner notification interventions in high-income countries were included in the review. The selection flowchart of the studies is presented in Fig. 1. The full-text articles excluded are detailed in Additional file 2. The characteristics of the included studies are in Table 1. The risk of bias assessment of the included studies is available in Additional file 3.

**Table 1:** Characteristics of the randomised controlled studies included in the review

Authors	Year	Settings	Participants	Interventions	Objectives	Outcomes	Data collection	Main results
Trent et al. [34]	2010	5 clinical sites US (Time frame not specified)	n = 126 girls, aged 15 years and older with CT or NG - Mean age: 17 years - Mean number of partners in the last 3 months: 1.7	• **Simple patient referral (n = 65)** • **Enhanced patient referral with video (n = 61)**	To examine the effectiveness of a brief behavioural intervention at the time of diagnosis of pelvic inflammatory disease, on subsequent behaviours of urban adolescents from an STI-prevalent community	• Proportion of index cases with ≥ 1 partner notified at 2 weeks of CT or NG diagnosis • Proportion of index cases with ≥ 1 partner treated at 2 weeks of CT or NG diagnosis • Proportion of index patients who abstained from intercourse at 2 weeks of CT or NG diagnosis	Face-to-face interview	• No significant difference between the two arms for the proportion of index cases with ≥ 1 partner notified • Index cases in the video arm are more likely to have ≥ 1 partner treated than those in the simple patient referral arm (aOR = 3.10 [1.03–9.39]) • No significant difference between the two arms for the proportion of index patients who abstained from intercourse
Wilson et al. [35]	2009	2 STI clinics US Jan 2002-Dec 2004	n = 600 index patients: 245 women and 355 men, with CT or NG - Mean age: 25 years − 96% were heterosexuals − 55% had ≥ 2 partners in the last 3 months	• **Enhanced patient referral with written information (n = 296)** • **Enhanced patient referral with counselling (n = 304)**	To assess the effectiveness of approaches targeting improved STI sexual partner notification through patient referral	• Proportion of index cases with ≥ 1 partner notified 1 month after CT or NG diagnosis • Proportion of index cases with positive CT or NG test at 6 months from initial diagnosis • Proportion of index cases with ≥ 1 unprotected anal or vaginal intercourse in the last 90 days at 6 months of CT or NG diagnosis	Face-to-face interview	• Index cases in the counselling arm are more likely to have ≥ 1 partner notified than those in the written information arm (aOR = 1.8 [1.02-3.0]) • Index cases in the written information arm are more likely to have a positive CT or NG test than those in the counselling arm (aOR = 2.2 (1.1–4.1]) • Index cases in the counselling arm are more likely to have no unprotected sexual intercourse than those in the written information arm (aOR = 1.5 [1.1–2.1])
Kissinger et al. [32]	2005	1 public STI clinic US Dec 2001-Mar 2004	n = 977 men, aged from 16 to 44 years with CT or NG − 48% were aged < 24 years − 68% had ≥ 2 partners at baseline - with 98% of women partners and 66% of casual partners	• **Simple patient referral (n = 285)** • **Enhanced patient referral with written information (n = 348)** • **Patient-delivered partner treatment (n = 344)**	To determine whether partner treatment is better than 2 partner referral methods in providing treatment to sex partners of men with CT or NG and in reducing recurrence of CT and NG	• Proportion of index cases whose partners said the treatment was taken at 4 weeks after CT or NG diagnosis • Proportion of index cases with positive CT or NG test 4 weeks after initial diagnosis	Face-to-face interview or CASI	• Compared to index cases in the simple patient referral arm, those in the written information arm are more likely to have partners with treatment taken (OR = 1.66 [1.22–2.27]), and even more for those in the partner treatment arm (OR = 2.88 [2.05–4.04]) • Compared to index cases in the simple patient referral arm, those in the partner treatment arm were less likely to have an NG or CT infection (OR = 0.38 [0.19–0.74], and even less likely for those in the written information arm (OR = 0.22 [0.11–0.44])
Golden et al. [33]	2005	1 public STI clinic US Sept 1998-Mar 2003	n = 2751 index patients: 646 heterosexual men and 2105 women, with either CT or NG or both - Mean age: 23 years - Mean number of partners in the last 2 months: 1.5	• **Simple patient referral (n = 1376)** • **Expedited partner treatment (n = 1375)**	To determine whether expedited partner treatment could reduce the rate of persistent or recurrent NG or CT infections among women and heterosexual men	• Proportion of index cases with all partners treated or tested negative at 3 to 9 weeks after CT or NG diagnosis • Proportion of index cases with persistent or recurrent CT or NG infection 3 to 9 weeks after initial diagnosis	Face-to-face interview	• Index cases in the partner treatment arm are more likely to have all their partners treated than those in the simple patient referral arm (RR = 1.2 [1.1–1.4]) • Persistent or recurrent infection is more likely to occur among index cases in the simple patient referral arm than in the partner treatment arm (RR = 0.76 [0.59–0.98])
Schillinger et al. [37]	2003	Family planning, adolescent, primary care, and STI clinics, emergency or hospital departments 5 US cities Sept 1996-June 2000	n = 1889 women, aged from 14 to 34 years, with CT − 83% were aged between 14–24 years - 82% had 1 partner in the last 2 months	• **Enhanced patient referral with written information (n = 943)** • **Patient-delivered partner treatment (n = 946)**	To determine whether repeated CT infections in women can be reduced with partner treatment compared with patient referral	• Proportion of index cases who gave treatment to ≥ 1 partner 1 month after CT diagnosis • Proportion of index cases who gave treatment to all partners 1 month after CT diagnosis • Proportion of index cases with positive CT test 4 months after initial diagnosis (follow-up at 1 month and 4 months)	Follow-up visit	• Index cases in the partner treatment arm are more likely to have given the treatment, than those in the written information arm are to have given the referral sheet to their only partner (85% versus 75%, p < 0.01), to at least one partner (81% versus 71%) and to all their partners (47% versus 25%) • No significant difference between the two arms for the proportion of index cases with a positive CT test
Cameron et al. [36]	2009	1 family planning clinic, 1 GUM clinic and 1 termination of pregnancy UK May 2004-Dec 2006	n = 330 women with CT, aged from 16 to 45 years - Mean age: 22 years − 37% had > 1 partner in the last 6 months	• **Enhanced patient referral with written information (n = 110)** • **Enhanced patient referral with self-sampling (n = 110)** • **Patient-delivered partner treatment (n = 110)**	To determine whether postal testing kit and expedited partner treatment reduced re-infection rates in women with uncomplicated CT infection over 12 months compared with patient referral	• Proportion of partners tested or treated 6 months after CT diagnosis • Proportion of index cases with positive CT test 12 months after initial diagnosis (follow-up at 3, 6, 9 and 12 months)	Telephone interview	• No significant difference in the proportion of partners confirmed tested or treated between the three arms • No significant difference in the proportion of reinfection between the three arms
Apoola et al. [39]	2009	An STI clinic UK Feb 2006-Mar 2007	n = 200 women with CT - Median age: 21 years − 99% were heterosexuals - Median number of identified partners per index case: 1 (1–1)	• **Enhanced patient referral with swab testing (n = 100)** • **Enhanced patient referral with urine testing (n = 100)**	To determine whether a better partner notification outcome could be achieved by giving female index patients with CT infection a home sampling kit instead of contact slips only	• Number of partners identified per index (median, IQR) • Number of traceable partners (median, IQR) • Number of partners treated in clinic (median, IQR) • Ratio of partners treated per index case • Number of partners treated within 28 days (median, IQR) • Number of index patients with ≥ 1 partner treated within 28 days per index case (%)	Face-to-face interview	• No significant difference between the two arms for all outcomes
Østergaard et al. [40]	2003	General practices in 4 counties Denmark Feb 1999-Mar 2000	n = 562 index patients: 414 women and 148 men, with CT - Mean age: 24 years - Mean number of partners in the last 12 months: 3	• **Enhanced patient referral with self-sampling (n = 304)** • **Enhanced patient referral with office sampling (n = 258)**	To compare the effectiveness of home sampling with office sampling for testing partners of men and women infected with CT	• Proportion of index cases with ≥ 1 partner tested for CT (time period not specified) • Proportion of index cases with ≥ 1 partner infected with CT (time period not specified)	Questionnaire	• The proportion of index cases with at least one partner tested was higher in the home sampling group than in the office group (in women RR = 2.2 [1.7–2.8], in men RR = 4.4 [2.2-9.0] • The proportion of index cases with at least one partner with CT was higher in the home sampling group than in the office group (in women RR = 1.63 [1.1–2.3], in men RR = 3.3 [1.2-9.0]
Low et al. [38]	2006	27 general practices UK Mar 2001-Oct 2002	n = 140 index patients: 92 women and 48 men, with CT − 90% were aged between 16–24 years - Mean number of partners per index case at baseline: 1.5	• **Referral to GUM clinic for partner notification interview with a specialist health advisor (n = 68)** • **Partner notification interview with a trained nurse on site (n = 72)**	To evaluate the effectiveness of a practice nurse-led strategy to improve the notification and treatment of partners of people with CT infection	• Proportion of index cases with ≥ 1 partner treated at 6 weeks of CT diagnosis • Proportion of index cases with all partners treated 6 weeks after CT diagnosis • Average number of partners treated per index case 6 weeks after CT diagnosis • Proportion of index cases with positive CT test 6 weeks after initial diagnosis	Telephone interview	• No significant difference between the two arms except for the proportion index cases with all partners treated higher in those in the nurse arm than in those in the GUM referral arm (51.4% versus 30.9%, p = 0.014)

aOR: adjusted odds ratio, CASI: computer-assisted self-interviewing, CT: Chlamydia trachomatis, GUM: genitourinary medicine, IQR: interquartile range, NG: Neisseria gonorrhoeae, OR: odds ratio, RR: risk ratio, STI: sexually transmitted infection, UK: United Kingdom, US: United States

**Simple patient referral:** Index patients were advised to tell their partners that they had been exposed to a sexually transmitted infection and to recommend that they get tested and treated.

**Enhanced patient referral with video:** Simple patient referral + Short video watched by index patients after their STI diagnosis.

**Enhanced patient referral with written information:** Simple patient referral + Written information to give to their partners (in the form of a leaflet, booklet or card containing information about the STI, the importance of getting tested and a list of clinics where they can obtain free care).

**Enhanced patient referral with counselling:** Simple patient referral + Written information to give to their partners + Counselling sessions for index patients at their STI diagnosis.

**Patient-delivered partner treatment or expedited partner treatment:** Simple patient referral + Written information to give to their partners + Medication to give to their sexual partners without consultation with a health professional. The medication package contains antibiotics, medication instructions, warnings about adverse effects.

**Enhanced patient referral with self-sampling:** Simple patient referral + Written information to give to their partners + Self-sampling kit to give to their partners. The kit contained materials to collect samples by the partner at home, instructions and a pre-paid envelope to send the sample to a laboratory.

**Enhanced patient referral with office sampling:** Simple patient referral + Written information to give to their partners + Office sampling kit to give to their partners. Partners were asked to bring the kit to a medical office for a healthcare provider to take a swab sample.

**Enhanced patient referral with swab testing:** Simple patient referral + Written information to give to their partners for testing by urethral swab and treatment at the clinic.

**Enhanced patient referral with urine testing:** Simple patient referral + Written information to give to their partners + Urine self-sampling kit to bring at the clinic and get treated.

The studies were published between 2003 and 2010, and conducted in the US (n = 5), UK (n = 3) and Denmark (n = 1), in STI clinics (n = 6), family planning clinics (n = 2), hospitals (n = 2) and general practices (n = 2). They targeted adolescent girls (n = 1), women (n = 7), or men (n = 5) with chlamydia, gonococcal infection or both. Participants enrolled were more likely to be < 25 years, heterosexual individuals with ≥ 2 sexual partners in the year. No randomised controlled trial targeting MSM, people living with HIV or evaluating partner notification of HIV or sexually transmitted HCV or syphilis were found since 2000.

The nine included articles reported patient referral assisted by two types of interventions: providing to index cases (1) information (via counselling sessions or video) to encourage them to inform their sexual partners (n = 3 studies), (2) something to give to their exposed sexual partners like written information on STI testing and prevention, self-testing kit or STI treatment (n = 7 studies). The interventions and control arms are detailed in Table 1.

Three studies compared simple patient referral to enhanced patient referral interventions, where the index patient (men [32], heterosexual men and women [33], girls [34] with chlamydia or gonorrhoea) was given additional information or tools to encourage partner notification (i.e. written information [32], expedited partner treatment [32, 33], brief educational video [34]). Compared to the simple patient referral, these enhanced interventions have been shown to be effective in terms of a higher proportion of index patients with at least one partner treated for the STI [32–34] and a lower proportion of reinfected index patients in the months following the intervention [32, 33].

Three other studies evaluated patient referral interventions enhanced with counselling [35], self-sampling kit [36], and partner treatment [37] compared to patient referral intervention enhanced with written information as a control arm (in women and men with chlamydia or gonorrhoea [35] and women with chlamydia [36, 37]). This time, no significant difference was observed between the two arms, in terms of tested or treated partners and reinfection rate in index patients [36, 37]. However, index patient counselling or expedited treatment improved the partners’ information and treatment delivery, respectively [35, 37].

The last three studies assessed the effectiveness of interventions designed to facilitate the access of index patients to STI partner notification services [38] or of notified partners to STI testing [39, 40]. One showed that partners were more likely to be treated when index patients (men and women) diagnosed with chlamydia in general practice received partner notification counselling by an on-site nurse, compared to when they were referred to a dedicated partner notification service [38]. In the two other studies, partners were given a sampling kit for STI testing by the index patients (women with chlamydia [39], men and women with chlamydia [40]). In one arm partners had to bring the kit to a healthcare provider to be tested, in the other arm, it was a self-sampling kit which partners had to drop off at the clinic [39] or return by post for analysis [40]. Only the self-sampling kit returned by post, presented as more convenient, increased the proportion of index cases with at least one partner tested [40].

In total, seven of nine studies concluded in favour of the experimented intervention. However, we observed a large heterogeneity in the intervention components and outcomes which did not allow us to have a meaningful overview of the partner notification process or transfer the interventions to other contexts and populations.

### Choice of outcomes

Among the nine identified randomised controlled trials on STI partner notification, 16 outcomes were counted regardless of the different times of measurement. Some addressed partner notification practices (e.g. the proportion of index patients with at least one partner informed, tested or treated), others, the STI circulation by index case reinfections, and others, participants’ sexual behaviour change after the intervention (Table 1). We counted a median of three outcomes per study (range: 2–6), most often two outcomes assessing the partner notification practices combined with another one assessing reinfection of index cases. The authors adopted a pragmatic posture to answer the question of the effectiveness of partner notification interventions, focusing on easier collected expected effects at the individual level. The related effects of partner notification interventions (behaviour changes, risk perception or adverse effects) were less likely to be explored.

In each of the nine studies included, at least one outcome regarding partner notification practices by index cases was found (Table 1). Most of the time (n = 5/9), this outcome was the proportion of index cases with at least one partner informed [34, 35], tested [40], infected [40] or treated [32, 34, 38], depending on the intervention studied. It was also the proportion of index cases with all partners treated [33, 38] or who gave the treatment to at least one partner or all partners [37], or the median number of index cases with at least one partner treated [39]. Other outcomes were used, such as the proportion of partners tested or treated [36] or the median number of partners identified, traceable and treated, or the ratio of partners treated per index case [39]. The choice of these outcomes was appropriate to (1) the objective of assessing the acceptability of index cases with the partner notification practices and the access of partners to testing and/or treatment; (2) the profiles of the participants involved in these studies, i.e. quite young heterosexual people with multiple partners. Although the definitions of multi-partner sex differ, relatively few partners were reported by index cases during months before their STI diagnosis, suggesting that few partners were exposed. The outcomes used, such as the proportion of index cases with at least one partner notified, tested or treated were adapted to the profiles of participants. Only one study, by Apoola et al. [39], evaluating the effectiveness of a partner notification intervention based on a self-sampling kit for STI testing given by index women with chlamydia to their partners versus written information encouraging testing in STI clinics, used as outcomes the median number of partners identified, contactable or treated per index case. No difference was found between the two arms. The authors could not conclude in favour of the self-sampling kit. However, since the median number of partners at baseline was equal to 1, both interventions were effective in treating partners.

In most studies (n = 6/9), the outcomes described above were combined with an outcome regarding reinfection of index cases [32, 33, 35–38]. Depending on the study, the reinfection rate was measured at 1, 2, 3, 4, 6 or 12 months after diagnosis. While the outcomes on the partner notification practices were related to the benefit of partner notification to sexual partners, reinfection reflects the impact of the partner notification process in terms of benefit to index cases. Furthermore, reinfection is a more global outcome that highlights the effect of the overall partner notification process.

Two studies investigated sexual and preventive behaviour following partner notification. One study addressed the issue of abstinence of index cases during the treatment of their STI [34] and another one the condom use of index cases following their STI diagnosis [35]. These outcomes were found in studies assessing behavioural interventions delivered to index cases, namely a brief behavioural video [34] and counselling [35]. These outcomes were collected in addition to other outcomes regarding partner notification practices and index case reinfection. Only one study collected data on deleterious effects related to partner notification (i.e. arguments, fight or physical violence) [35].

Each outcome provided different information on the notification process without really describing it completely. The outcomes that most often (n = 7/9 studies) lead to a conclusion about the intervention were those relating to partner notification practices [32–35, 37, 38, 40]. In studies where the effect on reinfections was also significant (n = 3/6 studies with both outcomes), both types of outcomes were consistent with a benefit of the intervention [32, 33, 35].

### Methods of outcomes collection

The method of data collection, i.e. how and from whom the data are collected, could lead to biases that might affect the conclusions of the studies.

Almost all studies (n = 8/9) used interviews with index cases to collect data on partner notification practices. The interviews were conducted face-to-face (n = 6/9) [32–35, 37, 39] or by telephone (n = 2/9) [36, 38]. In two studies, the interviews were carried out by an independent investigator, not involved in the intervention or care of the participants [35, 38]. In the other six studies, the interviews were carried out by a staff member involved in the recruitment of participants or the delivery of the intervention, accentuating the potential social desirability bias which may overestimate the effect of the intervention [32–34, 36, 37, 39]. Participants who fail to notify their partners may be less likely to answer questions or tell the truth.

Only one study used a self-administered questionnaire to collect data from index cases [40]. This method of data collection limits the social desirability bias that can be induced by a face-to-face or telephone interview. However, depending on the outcomes and populations, self-administered questionnaires could be less appropriate than other routes. Several complementary collection routes could be used. In their study, Østergaard et al. [40] used a self-administered questionnaire to collect data on the characteristics of the index cases, such as previous STI and sexual behaviour that could suffer from desirability bias, but not the partner notification outcomes, which need to be accurate and complete. The outcomes, which were the proportions of index cases who had at least one partner tested, and tested positive for chlamydia, were collected directly from the study laboratory where the partners had to send their urine or vaginal samples for analysis. Another example shows the benefits of the interview. Trent et al. [34] evaluated the effectiveness of a partner notification informational video in adolescent girls with chlamydia or gonococcal infection, using a face-to-face interview to collect data. Interviewers were experienced in locating community members exposed to STIs and had previously monitored adolescents from this community. This method may have helped to build the girls’ confidence and enabled the collection of a wider range of data (e.g. girls’ treatment uptake, complications, partner notification, partner treatment and abstinence during the treatment period). In this context, the interview maximised girls’ participation and minimised missing data. Authors have to find a balance in data collection between completeness and quality of the data obtained.

The partner notification process is composed of a succession of actions between the intervention on the index case and the testing and/or treatment of the notified partners, which imposes the collection of the outcome a few weeks after the intervention. This inevitably results in dropouts and a possible attrition bias resulting in differences between participants who complete the follow-up visit and those who do not. To limit these drop-outs, one study collected data at the follow-up visit, which requires participants to return to the centre only once. This was done in the study by Trent et al. [34]. However, only 62% of the girls returned for follow-up two weeks after their STI diagnosis. Two studies used a follow-up by telephone [36, 38], an active method of reaching participants, but this is contingent upon success in contacting the participants (e.g. wrong number, voicemail, call filtering). This telephone follow-up to assess partner notification outcomes provided data from 74% of index cases at 6 weeks of STI diagnosis in the study of Low et al. [38] and 44% of index cases at 6 months in the study of Cameron et al. [36]. The outcome the most affected by drop-outs was the reinfection rate in index patients, because it was measured several weeks to months later. Furthermore, it requires participants to provide urine or swab samples, and they might be less compliant than when providing data. This was illustrated by the study by Kissinger et al. [32], in which 80% of the index men completed the follow-up visit at one month, but only 38% of them provided the urine sample needed to assess reinfection. These authors compared participants who gave a urine sample with those who did not. They found no differences in social and demographic characteristics, suggesting that those who did not were less compliant with the intervention. They were the only ones to conduct such comparisons.

The assessment of the effectiveness of partner notification is based essentially on the reports of index cases. While this is most relevant for the outcomes related to index cases (e.g. proportion of index cases with ≥ 1 partner notified, proportion of index cases reinfected at 1 month), the information related to sexual partners (i.e. proportion of index cases with ≥ 1 partner tested or treated) was also reported by index cases [32–34, 36–40]. In no study were the partners interviewed. Index cases seeking information from their sexual partners may lead to differential information bias. Kissinger et al. [32] observed that index men in the patient-delivered partner treatment arm were more likely to have checked whether their partners had taken the treatment than those in the simple patient referral arm.

### Interpretation of outcomes

Based on the studies included in the review, we have identified factors, methodological or contextual, that need to be considered when interpreting the results.

The succession of intervention components in the notification process plays an important role in interpreting the outcomes. For example, the proportion of index cases with at least one partner treated (n = 2 studies) was interpreted differently according to the intervention under study: information provided to index cases by an educational video [34] versus treatment delivered to index cases to be given to partners [38]. The same outcome was measured, the partner treatment, which occurred at different steps in the partner notification process depending on the intervention. In the first study, partner treatment is the result of a more complex process than when it follows immediate partner access to treatment. Intermediate outcomes would help to understand the acceptability of each step of the process.

One contextual factor which could affect partner notification practices and influence the interpretation of outcomes was the type of partner. One of the nine studies addressed it: Kissinger et al. [32] evaluated the effectiveness of partner notification enhanced with written information in one arm and expedited partner treatment in another arm, versus simple patient referral in men with chlamydia or gonorrhoea. Both interventions were shown to be effective in terms of the proportion of index patients whose notified partners told them that the treatment was taken. However, by assessing the outcome by type of partner, the authors showed that index cases (all arms together) were more likely to report that their partners had taken the treatment when it was the main partner rather than casual partners. In the present case, the results as they were presented did not let us decide between a greater effectiveness of the intervention in the main partners or a greater proximity that made it easier for the index cases to be informed about the treatment of the main partners. The type of partner would be interesting to consider when transferring the intervention to other populations.

The number of exposed partners per index case should be considered in order to assess whether or not the intervention was effective, particularly in studies where an outcome like “at least one partner” was used. If at least one partner was notified while few partners were exposed, as in the included studies [34, 35, 37–39], we could conclude that the intervention was effective. This outcome could not be interpreted in this way if the numbers of exposed partners were high.

The time point chosen to measure outcomes was important for interpreting the results. While the time chosen for measuring partner testing or treatment was around 1 month, we observed more variability when assessing reinfections of index cases. The cause of reinfections can be difficult to interpret, as Kissinger et al. [32] mentioned: it was “*impossible to determine whether the follow-up infections were reinfections with strains from original partners, new infections with strains from newly acquired partners, or persistence of the original infection*”. The probability of occurrence of each reinfection cause is time-dependent and differs if the reinfection occurs 1 month [32] or 12 months [36] after the initial diagnosis. Cameron et al. [36] measured reinfections at different time points (3, 6, 9 and 12 months) and demonstrated that most reinfections (n = 21/34) occurred within 6 months following the STI diagnosis. Intervention failure, i.e. reinfection by the initial partner who was not treated, represented 26% of reinfections regardless of the arm (no significant difference in the proportion of reinfections between arms). Golden et al. [33] found that, compared to those in the simple patient referral arm, index cases in the partner treatment arm were less likely to have reinfection in following weeks. In addition to failure to treat sex partners, they identified behavioural factors significantly associated with a higher risk of reinfection: having sex with an untreated partner and the number of partners with whom condoms were not used since treatment. These two studies showed that reinfections were influenced by factors depending on sexual behaviour of the index patient and times of measurement. Except when the partner notification intervention contained counselling for the index case [35], we would expect these factors to impact similarly on the different arms of the trial. This would explain why the reinfection outcome leads less often than the partner notification practice outcomes to a decision in favour of the intervention (50% vs. 78% of the studies).

The partner testing and treatment outcomes should be interpreted with regard to the proportion of partners who already knew that they were infected with the STI and might be tested and treated outside of the study. This point was only addressed in the study by Østergaard et al. [40], where 3% of the index cases had one partner who was already aware of their chlamydia infection. The authors excluded them from the analysis. Not considering the proportion of partners who know they are already infected before being notified may lead to an underestimation of the effectiveness of the partner notification intervention.

## Discussion

Complexity in health interventions has already been described, highlighting methodological challenges when evaluating these interventions [41, 42]. Partner notification of STIs is a complex intervention and we have seen from this review of randomised controlled trials in high-income countries since 2000 that to assess its effectiveness we need to identify appropriate outcomes, find complementary ways of collecting them, and pay attention to all contextual factors in their interpretation.

To be effective, a partner notification intervention has to be acceptable for both the index cases (to actually inform or give treatment to their exposed partners, for example) and the notified partners (to actually get tested and/or take the treatment). Through the nine studies included in this review, we observed a large number of outcomes (n = 16) used to assess partner notification effectiveness. Several outcomes were found in several studies, with generally one or two outcomes focused on partner notification practices associated with an outcome reflecting the circulation of STIs. Most of the outcomes were appropriate for measurement, in the studied populations, of the main expected effects of the interventions: informing of the partners by index cases, and testing and/or treatment of the notified partners. However, the partner notification process is a succession of intervention components. As we have shown, according to the intervention, the same outcome may be interpreted differently depending on when the event it measured occurred in the partner notification process. To understand better why an intervention works or not, data should be collected at each step of the process and some additional outcomes deserve to be investigated to transfer and adapt the intervention to other contexts [42–46]. Additional data on populations, interventions, control groups and contexts were missing to evaluate feasibility of the intervention. Very few studies considered outcomes on sexual behaviour of index patients that could have changed after receiving a partner notification intervention. These behaviours deserve to be studied further, in particular by in-depth qualitative surveys [42].

Reinfection of index cases provides information on a more global effect of partner notification. However, it is highly influenced by the time of measurement and the sexual behaviour of the study population. The decision on whether or not the intervention is effective should not be based on this outcome alone. However, reinfection assessed at the individual level of index cases may give an indication of STI transmissions at the community level.

The review did not study the impact of partner notification interventions on STI epidemics but, at a first step, remained focused on randomised controlled trials conducted at the individual-level. When implementing a new intervention in a given population, as addressed above, it is important to have evidences of the effectiveness at an individual-level, be able to identify what is not feasible, not acceptable, etc. In a second step, the evaluation of the partner notification effectiveness at the population-level may be considered. This is what Golden et al. have done to assess the effectiveness of expedited treatment of sexual partners to reduce the rates of gonococcal or chlamydial reinfection in heterosexual men and women in public STI clinic in US. They first conducted a randomised controlled trial evaluating the effectiveness of the intervention at the individual-level (Golden et al. 2005, included in this review), then the intervention was scaled-up to the community and evaluated by a cluster randomised trial with outcomes collected at the population-level (Golden et al. 2015 [47]). The outcomes used and methods to collect them differed in the two studies: the proportion of index cases with chlamydial or gonococcal reinfection collected directly from index cases versus the positivity rate of chlamydial tests carried out by sentinel clinics and the incidence rate of gonorrhoea reported by local health jurisdictions. To our knowledge, the study of Golden et al., published in 2015, was the only study assessing a partner notification intervention at the population-level in high-income countries. Although it assessed the effectiveness of the same intervention than those published in 2005, their outcomes and methods of both studies were not comparable and this study could not be included in the review.

The studies included in our review were all carried out in homogeneous populations: young heterosexual people diagnosed with chlamydia or gonococcal infection and described as having at least two partners during the preceding year. The limited data available on partners suggested that they had few concurrent partners. The outcome found in several studies was the proportion of index cases with “at least one partner” who was informed, tested or treated. Given the population studied, this type of outcome was appropriate. While outcomes based on the median number or the proportion of partners, as seen in the study published by Apoola et al. [39], would more probably be appropriate in studies carried out among individuals with many concurrent partners, or consecutive partners over a long period of exposure as for HIV [18]. Furthermore, a better description of index cases and their partners might help to identify populations for whom the intervention was effective and those for whom it was not.

In the situation where many partners could have been exposed, it is important to consider the type of partner (main, new casual, one-off partner or sex worker). This is in line with recent recommendations on optimising outcomes for partner notification services in the UK [48]. A study conducted in France among MSM on PrEP [49] showed that the main partner was more likely to be notified than casual partners. However, in the case of concurrent main and casual partnerships, main partners were less likely to be notified than casual partners, for fear of disclosure. Considering the type of partner in assessing partner notification effectiveness would help adapt the proposed interventions.

The assessment of partner notification interventions and their processes is limited by the collection of data on partners from index cases. No study included in the review collected information directly from partners, and eight out of nine collected partners’ information from the index patients. The non-representation of notified partners in studies may be related to the challenge of recruiting index cases and their sexual partners. Including partners in the studies would select partners, but it may provide more reliable and less biased data on the outcomes related to testing or treatment by the notified partners, which justifies the whole notification process. The few studies that enrolled people who were notified and sought STI testing were qualitative [50, 51]. This type of study enables the exploration of concepts such as experience, intention to use, barriers and levers to partner notification, as well as the investigation of the a priori acceptability of partner notification intervention.

From the perspective of replicating or designing a new intervention, the type of STI could have an impact on the notification practices due to the retrospective period for searching for exposed partners, varying from days to months [52], and the populations they affect. These two factors affect the number of exposed partners, the proportions of one-off or anonymous partners who cannot be contacted, and the proportion of partners who are already aware of their infection before being notified. Characteristics of STIs and populations should be kept in mind when defining and interpreting the partner notification outcomes. However, the studies that met inclusion criteria, i.e. randomised controlled trials on partner notification effectiveness published over the period 2000 to 2021 in high-income countries, did not assessed all STIs. They were conducted among people diagnosed with chlamydia and/or gonococcal infection. None assessed HIV- or sexually transmitted HCV- or syphilis-specific partner notification interventions. Likewise, the findings on the effectiveness of partner notification interventions cannot be extrapolated to low- and middle-income countries.

The study protocol was not registered on PROSPERO as recommended. However, the review was monitored by a steering committee, which ensured that the review was conducted in accordance with the study protocol and that there were no flaws in the article selection process. The research strategies are detailed in Additional file 1.

To our knowledge, three reviews have been published on the effectiveness of partner notification [21–23]. The review published by Trelle et al. in 2007, covers partner notification interventions for STIs up to 2005 [21], and the review published by Dalal et al. in 2017, covers partner notification interventions for HIV up to 2016 [23], both in high-, middle- and low-income countries. Our study included no other study already included in both of these reviews. Another review on partner notification interventions for STIs, including HIV, also conducted in high-, middle- and low-income countries was published by Ferreira et al. in 2013 [22]. All studies included in our review were found in this review. But the review also analysed seven studies carried out in high-income countries before 2000 when STI epidemics were lower than after 2000 and highly effective antiretroviral treatment for HIV were not available yet. This review did not identify a single optimal strategy for partner notification. Although it showed that expedited partner therapy and enhanced patient referral were more effective than simple patient referral for STIs causing urethritis and cervicitis. Our restriction to more homogeneous contexts was intended to try to identify more specific interventions. Our results confirm that providing index cases with something to give to partners (written information, treatment) is a factor that can improve partner notification. However, we have gone further by assessing the heterogeneity of the outcomes and providing guidance to help choose, collect and interpret these outcomes in a context of complex intervention like partner notification.

## Conclusion

Throughout a review of STI partner notification interventions, we highlighted the importance of the choice of outcomes for such complex interventions. We suggested collecting outcomes at each step of the partner notification process, additional data on the participants at each level (index cases and their partners) and contextual factors affecting the process. These insights would help to understand why and under what conditions the intervention is considered effective and therefore can be replicated or adapted to other populations and contexts.

### Electronic supplementary material

Below is the link to the electronic supplementary material.

Supplementary Material 1

## Data Availability

All relevant data is included in the manuscript. The protocol of the review is available on request from the corresponding author.

## Abbreviations

HCV: Hepatitis C virus
HIV: Human immunodeficiency virus
LGV: Lymphogranuloma venereum
MSM: Men who have sex with men
STI: Sexually transmitted infection
