# Supplementary material for: Assessing complex interventions: a systematic review of outcomes used in randomised controlled trials on STI partner notification in high-income countries
Source: BMC Public Health. 2023 Sep 21;23:1838. doi: 10.1186/s12889-023-16763-9 (PMC10512513; doi:10.1186/s12889-023-16763-9)
Supplement: Supplementary file 1 — Supplementary Material 1 [file 12889_2023_16763_MOESM1_ESM.docx]

**Additional file 1. Search strategies**

**Medline search strategy**

| **#1** | **Contact tracing[Title] OR Contract referral[Title] OR Enhanced patient referral[Title] OR Partner counseling and referral services[Title] OR Partner management[Title] OR Partner notification*[Title] OR Partner services[Title] OR Provider referral[Title] OR Contact tracing[MeSH Major Topic]** |
| --- | --- |
| **#2** | **Expedited partner therapy[Title] OR Patient delivered partner therapy[Title] OR Patient delivered partner treatment[Title] OR Patient delivered therapy[Title] OR Accelerated partner therapy[Title]** |
| **#3** | **#1 OR #2** |
| **#4** | **Sexually transmissible disease*[Title/Abstract] OR Sexually transmissible disorder*[Title/Abstract] OR Sexually transmissible infection*[Title/Abstract] OR Sexually transmissible infectious disease*[Title/Abstract] OR Sexually transmitted disease*[Title/Abstract] OR Sexually transmitted disorder*[Title/Abstract] OR Sexually transmitted infection*[Title/Abstract] OR Sexually transmitted infectious disease*[Title/Abstract] OR STD[Title/Abstract] OR STDs[Title/Abstract] OR STI[Title/Abstract] OR STIs[Title/Abstract] OR VD[Title/Abstract] OR Venereal disease*[Title/Abstract] OR Venereal disorder*[Title/Abstract] OR Venereal infection*[Title/Abstract] OR Syphilis[Title/Abstract] OR Chlamydia[Title/Abstract] OR Gonorrhea*[Title/Abstract] OR Neisseria gonorrhoeae[Title/Abstract] OR sexually transmitted diseases[MeSH Terms] OR syphilis[MeSH Terms] OR Chlamydia[MeSH Terms] OR Chlamydia infection[MeSH Terms] OR Gonorrhea[MeSH Terms]** |
| **#5** | **Hiv[Title/Abstract] OR Hiv1[Title/Abstract] OR Hiv2[Title/Abstract] OR Human immunodeficiency virus[Title/Abstract] OR Acquired immunodeficiency syndrome[Title/Abstract] OR Hiv[MeSH Terms] OR Infection, hiv[MeSH Terms]** |
| **#6** | **Sexually transmitted hepatitis c[Title/Abstract] OR Sexually acquired hepatitis c[Title/Abstract] OR (HCV[Title/Abstract] AND MSM[Title/Abstract])** |
| **#7** | **#4 OR #5 OR #6** |
| **#8** | **#3 AND #7** Filters: **Randomized Controlled Trial, from 2000/1/1 - 2021/12/31** |

**Cochrane Library search strategy**

| **#1** | **((Contact tracing) OR (Contract referral) OR (Enhanced patient referral) OR (Partner counseling and referral services) OR (Partner counselling and referral services) OR (Partner management) OR (Partner notification*) OR (Partner services) OR (Provider referral)):ti** |
| --- | --- |
| **#2** | **MeSH descriptor: [Contact Tracing] explode all trees** |
| **#3** | **((Expedited partner therapy) OR (Patient delivered partner therapy) OR (Patient delivered partner treatment) OR (Patient delivered therapy) OR (Accelerated partner therapy)):ti** |
| **#4** | **#1 OR #2 OR #3** |
| **#5** | **((Sexually transmissible disease*) OR (Sexually transmissible disorder*) OR (Sexually transmissible infection*) OR (Sexually transmissible infectious disease*) OR (Sexually transmitted disease*) OR (Sexually transmitted disorder*) OR (Sexually transmitted infection*) OR (Sexually transmitted infectious disease*) OR (STD) OR (STDs) OR (STI) OR (STIs) OR (VD) OR (Venereal disease*) OR (Venereal disorder*) OR (Venereal infection*) OR (Syphilis) OR (Chlamydia) OR (Gonorrhea*) OR (Neisseria gonorrhoeae)):ti,ab** |
| **#6** | **MeSH descriptor: [Sexually Transmitted Diseases] 5 tree(s) exploded** |
| **#7** | **MeSH descriptor: [Syphilis] 7 tree(s) exploded** |
| **#8** | **MeSH descriptor: [Chlamydia] explode all trees** |
| **#9** | **MeSH descriptor: [Gonorrhea] 6 tree(s) exploded** |
| **#10** | **((Hiv) OR (Hiv1) OR (Hiv2) OR (Human immunodeficiency virus) OR (Acquired immunodeficiency syndrome)):ti,ab** |
| **#11** | **MeSH descriptor: [HIV] explode all trees** |
| **#12** | **((Sexually transmitted hepatitis c) OR (Sexually acquired hepatitis c) OR ((HCV) AND (MSM))):ti,ab** |
| **#13** | **#5 OR #6 OR #7 OR #8 OR #9 OR #10 OR #11 OR #12** |
| **#14** | **#4 AND #13 with Cochrane Library publication date Between Jan 2000 and Dec 2021, in Trials** |
| **#15** | **((randomized rontrolled trial) OR (randomised controlled trial)):ti,ab** |
| **#16** | **#14 AND #15** |

**Embase search strategy**

| **#1** | **'partner notification'**:ti,ab,kw OR **'partner notification'**/exp OR **'partner notification'** OR ((**'disease notification'**:ti,ab,kw OR **'disease notification'**/exp OR **'disease notification'**) AND (**spouse**:ti,ab,kw OR **'spouse'**/exp OR **'spouse'** OR **wife**:ti,ab,kw OR **husband**:ti,ab,kw OR **'domestic partner'**:ti,ab,kw OR **'intimate partner'**:ti,ab,kw OR **'sexual partner'**:ti,ab,kw OR **'partner management'**:ti,ab,kw)) OR **'contract referral'**:ti,ab,kw OR **'enhanced patient referral'**:ti,ab,kw OR **'partner counseling and referral services'**:ti,ab,kw OR **'partner management'**:ti,ab,kw OR **'provider referral'**:ti,ab,kw OR **'expedited partner therapy'**:ti,ab,kw OR **'expedited partner therapy'**/exp OR **'expedited partner therapy'** OR **'patient delivered partner therapy'**:ti,ab,kw OR **'patient delivered partner therapy'**/exp OR **'patient delivered partner therapy'** OR **'patient delivered partner treatment'**:ti,ab,kw OR **'accelerated partner therapy'**:ti,ab,kw OR **'patient delivered partner medication'**:ti,ab,kw OR **'patient delivered therapy'**:ti,ab,kw |
| --- | --- |
| **#2** | **'sexually transmitted disease'**/exp OR **'chlamydiasis'**/exp OR **'human immunodeficiency virus infection'**/exp OR ((**'sexual transmission'**/exp OR **'men who have sex with men'**/exp) AND **'hepatitis'**/exp) |
| **#3** | **#1** AND **#2** |
| **#4** | **#3** AND [2000-2021]/py |
| **#5** | **#4** AND **'randomized controlled trial'**/de |
| **#6** | **#5** AND [embase]/lim NOT ([embase]/lim AND [medline]/lim) |

**Web of Science search strategy**

| **#1** | **TI=(“contact tracing” or “contract referral” or “enhanced patient referral” or “partner counseling and referral services” or “partner management” or “partner notification” or “partner services” or “provider referral” )** |
| --- | --- |
| **#2** | **TI=(“expedited partner therapy” or “patient delivered partner therapy” or “patient delivered partner treatment” or “patient delivered therapy” or “accelerated partner therapy”)** |
| **#3** | **#1 or #2** |
| **#4** | **AB=(“sexually transmissible disease” or “sexually transmissible disorder” or “sexually transmissible infection” or “sexually transmissible infectious disease” or “sexually transmitted disease” or “sexually transmitted disorder” or “sexually transmitted infection” or “sexually transmitted infectious disease” or “std” or “sti” or “vd” or “venereal disease” or “venereal disorder” or “venereal infection” or “syphilis” or “chlamydia” or “gonorrhea” or “neisseria gonorrhoeae”)** |
| **#5** | **AB=(“hiv” or “human immunodeficiency virus” or “acquired immunodeficiency syndrome”)** |
| **#6** | **AB=(“sexually transmitted hepatitis c” or “sexually acquired hepatitis c” or (“hcv” and “msm”) )** |
| **#7** | **#4 OR #5 OR #6** |
| **#8** | **#3 AND #7** |
| **#9** | **AB=("randomized controlled trial" or "randomised controlled trial")** |
| **#10** | **#8 and #9 Timespan: 2000-01-01 to 2021-12-31** |

**CINAHL search strategy**

| **#1** | **TI “contact tracing” or “contract referral” or “enhanced patient referral” or “partner counseling and referral services” or “partner management” or “partner notification” or “partner services” or “provider referral”** |
| --- | --- |
| **#2** | **TI “expedited partner therapy” or “patient delivered partner therapy” or “patient delivered partner treatment” or “patient delivered therapy” or “accelerated partner therapy”)** |
| **#3** | **S1 OR S2** |
| **#4** | **AB “sexually transmissible disease” or “sexually transmissible disorder” or “sexually transmissible infection” or “sexually transmissible infectious disease” or “sexually transmitted disease” or “sexually transmitted disorder” or “sexually transmitted infection” or “sexually transmitted infectious disease” or “std” or “sti” or “vd” or “venereal disease” or “venereal disorder” or “venereal infection” or “syphilis” or “chlamydia” or “gonorrhea” or “neisseria gonorrhoeae”** |
| **#5** | **AB “hiv” or “human immunodeficiency virus” or “acquired immunodeficiency syndrome”** |
| **#6** | **AB “sexually transmitted hepatitis c” or “sexually acquired hepatitis c” or (“hcv” and “msm”)** |
| **#7** | **S4 OR S5 OR S6** |
| **#8** | **S3 AND S7** |
| **#9** | **AB "randomised controlled trial" or "randomized controlled trial" or "rct"** |
| **#10** | **S8 AND S9** |
| **#11** | **S8 AND S9 Date de publication: 20000101-20211231** |

**Additional file 2. Studies excluded from the review after the full-text review (n=3)**

| **Authors and year** | **Participants** | **Interventions and sample sizes by study arm** | **Objectives** | **Outcomes** | **Reasons of study exclusion** |
| --- | --- | --- | --- | --- | --- |
| Escourt et al., 2015  *(pilot RCT)* | n=199 women, aged ≥16 years old, with CT | - Accelerated partner-therapy via hotline (n=68) - Accelerated partner-therapy via pharmacy (n=65) - Control: Standard patient referral (n=66) | Primary objective:  To assess the feasibility and acceptability of accelerated partner-therapy  Secondary objective:  To provide preliminary evidence of effectiveness of accelerated partner-therapy | - Proportion of partners known to have been treated within 6 weeks of index diagnosis - Number of partners treated per index patient - Number of partners notified per index patient - Time to partner treatment - CT reinfection rate in index patients, 4–6 weeks after treatment | The sample set was not large enough to provide sufficient statistical power to meet this second objective. |
| Kerani et al., 2011 | n=75 MSM, aged ≥18 years old, with CT and/or NG | - Web-based partner notification via InSPOT (n=17) - Patient-delivered partner therapy (PDPT) (n=16) - InSPOT/PDPT (n=24) - Control: Simple patient referral (n=18) | Primary objective:  To determine whether PDPT and/or inSPOT would increase partner notification and/or treatment | - Number of partners notified per index patient - Number of partners treated per index patient - Number of partners tested for HIV per index patient - Number of partners tested for syphilis per index patient | The study was stopped prematurely due to too few inclusions and did not lead to conclusive results. |
| Tomnay et al., 2006 | n=105 men and women, aged ≥16 years old, CT or NGU | - Simple patient referral with written information, with the addition of websites addresses for STI clinic and STI (n=73) - Control: Simple patient referral with written information (n=32) | Primary objective:  To assess the acceptability of the addition of websites addresses on the standard letter used for partner notification  Secondary objective:  To determine if the addition of websites addresses would enhance the outcomes of partner notification | - Partners’ objection to the letters with the web address reported by index patients - Proportion of index patients with all partners notified - Proportion of index patients with ≥1 partner notified - Number of partners notified per index patient - Reinfection rate of CT or NGU in index patients, in the 2-12-week period post-treatment | The sample set was not large enough to provide sufficient statistical power to meet this second objective. |

CT: chlamydia trachomatis, NG: neisseria gonorrhoea, MSM: men who have sex with men, NGU: non-gonococcal urethritis

**References**

Estcourt CS, Sutcliffe LJ, Copas A, et al. (2015) Developing and testing accelerated partner therapy for partner notification for people with genital Chlamydia trachomatis diagnosed in primary care: a pilot randomised controlled trial. *Sexually transmitted infections* 91(8): 548–554. DOI: [10.1136/sextrans-2014-051994](https://doi.org/10.1136/sextrans-2014-051994).

Kerani RP, Fleming M, DeYoung B, et al. (2011) A randomized, controlled trial of inSPOT and patient-delivered partner therapy for gonorrhea and chlamydial infection among men who have sex with men. *Sexually transmitted diseases* 38(10): 941–946. DOI: [10.1097/OLQ.0b013e318223fcbc](https://doi.org/10.1097/OLQ.0b013e318223fcbc).

Tomnay JE, Pitts MK, Kuo TC, et al. (2006) Does the Internet assist clients to carry out contact tracing? A randomized controlled trial using web-based information. *International journal of STD & AIDS* 17(6): 391–394. DOI: [10.1258/095646206777323391](https://doi.org/10.1258/095646206777323391).

**Additional file 3. Risk of bias assessment of included studies**

The Cochrane Collaboration’s tool^[[1]](#footnote-1)^ provides a framework for considering the risk of bias of an intervention effect reported from a randomised trial. The effect assessed is a comparison of experimental and comparator interventions, for a specific outcome. The tool is structured into five domains that cover all types of biases that can affect results of randomised trials: randomisation process, deviations from the intended interventions, missing outcome data, measurement of the outcome and selection of the reported result.

For each domain, the tool lists standardised questions to help identifying the presence of bias. The tool also provides detailed criteria to objectively assess the level of risk (low, high, unclear) for each bias.

The risk of bias assessment for each outcome of the nine included study is presented in the following Table.

The main concerns about the outcomes used in the included studies relate to missing outcome data, outcome measurement and the selection of reported outcomes.

Missing outcome data (D3)

Four studies had one or more outcomes with an attrition rate >20%, that were therefore considered to have a high risk due to missing outcome data (Golden 2005, Kissinger 2005, Cameron 2009 and Trent 2010). However, in the study that compared the characteristics of participants who completed the follow-up and those who did not, no significant differences were found (Kissinger 2005). Therefore, the selection bias was minimized.

Measurement of the outcome (D4)

In two studies, the outcome assessment was not blinded, especially for primary outcomes, and could have led to a detection and information biases (Kissinger 2005 and Apoola 2009). We discussed this bias in the manuscript.

Selection of the reported result (D5)

For one study, the intervention arms reported in the protocol differed from those reported in the published article (Wilson 2009). A planned third arm corresponding to a third intervention was not carried out or reported. The outcomes could have suffered from a bias of selection in the reporting of the result. However, the study comparing the two arms was good quality and could be included into the review.


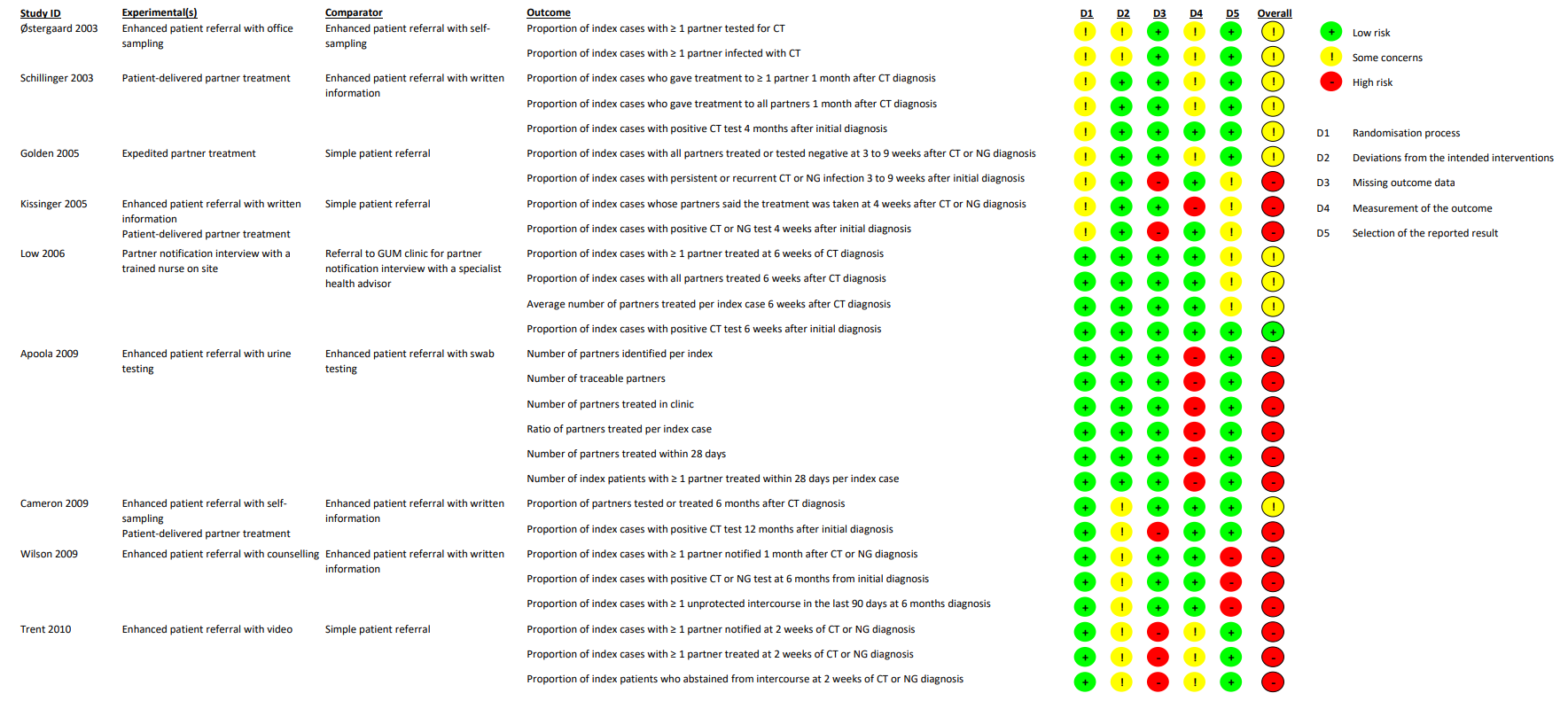


1. Sterne JAC, Savović J, Page MJ, Elbers RG, Blencowe NS, Boutron I, *et al.* **RoB 2: a revised tool for assessing risk of bias in randomised trials**. *BMJ* 2019; **366**:l4898. [↑](#footnote-ref-1)
